# Knowledge, attitudes, and practices toward xylometazoline nasal spray use among adults in Saudi Arabia: a cross-sectional survey

**DOI:** 10.3389/fphar.2026.1818680

**Published:** 2026-05-12

**Authors:** Fahad H. Baali, Abdullah A. Alshehri, Raghad D. Alsufyani, Hawazin S. Alsufyani, Wadia S. Alruqayb, Abdulkarim Alshammari, Saeed Saad A. Alghamdi, Mohra Aladwani, Fahad M. Alqarni, Ali H. Alwadai, Wael Y. Khawagi

**Affiliations:** 1 Department of Clinical Pharmacy, College of Pharmacy, Taif University, Taif, Saudi Arabia; 2 College of Pharmacy, Taif University, Taif, Saudi Arabia; 3 Department of Pharmacy Practice, College of Pharmacy, Northern Border University, Rafha, Saudi Arabia; 4 Pharmaceutical Outcom Department, Quality and Outcomes Monitoring, Branch of Ministry in Taif, Taif, Saudi Arabia; 5 Department of Clinical Pharmacy, College of Pharmacy, Najran University, Najran, Saudi Arabia

**Keywords:** awareness, nasal decongestants, OTC medicines, public health, Saudi Arabia, xylometazoline

## Abstract

**Background:**

Nasal congestion is a frequent symptom of upper respiratory illnesses. Over-the-counter decongestants such as xylometazoline are widely used, providing rapid relief but prone to misuse. In Saudi Arabia, there is limited evidence of public attitudes, practices, and predictors. This study aimed to assess knowledge, attitudes, and practices toward xylometazoline among adults in Saudi Arabia and to identify sociodemographic predictors of appropriate use.

**Methods:**

A cross-sectional survey, conducted between May and July 2025, included adults (≥18 years) residing in Saudi Arabia. A validated questionnaire was used to collect sociodemographic data, xylometazoline use, and assess KAP scores. Descriptive statistics summarized outcomes. Spearman’s rho assessed correlations among KAP domains. Predictors of knowledge, attitude, and practice were examined using binary logistic regression, reported as adjusted odds ratios (aORs) with 95% confidence intervals (CIs). Two-sided p < 0.05 was considered statistically significant.

**Results:**

A total of 422 individuals participated; 226 (53.6%) were female, and 185 (43.8%) were aged 18–30 years. Xylometazoline use in the past 12 months was reported by 295 (69.9%). Overall, 217 (51.4%) demonstrated good knowledge, 232 (55.0%) held a positive attitude, and only 37 (8.8%) reported good practice, while 187 (44.3%) reported poor practice. Postgraduate education was significantly associated with good knowledge (aOR 2.78, 95% CI 1.42–5.45), while chronic nasal infections with severe dryness were strongly associated with good practice (aOR 6.29, 95% CI 1.72–22.97). Good practice decreased with higher frequency of use (1–3 times/year: aOR 0.37; 4–6: aOR 0.18; >6: aOR 0.05; all p < 0.001). Regional variations in knowledge and attitudes were also observed.

**Conclusion:**

Xylometazoline use is highly prevalent in Saudi Arabia, with favorable knowledge and attitudes but suboptimal practices. Younger age and higher education were associated with better knowledge, while female sex and nasal conditions predicted safer practices. Public education and pharmacist-led counseling, particularly on safe duration of use, are essential to close the knowledge–practice gap and reduce misuse.

## Introduction

Nasal congestion is a common symptom of respiratory illnesses such as sinusitis, allergic rhinitis, and the common cold, and is a frequent cause of patient discomfort worldwide (Stewart et al., 2010; Chandra et al., 2009; Naclerio et al., 2010). It contributes to breathing difficulties, impaired sleep, and reduced daily functioning, all of which significantly affect quality of life (Meltzer et al., 2004). Globally, nearly one-third of adults report experiencing nasal obstruction, making it one of the most troublesome symptoms of upper respiratory tract conditions (Meltzer et al., 2004; Savouré et al., 2022). Beyond its clinical impact, persistent nasal congestion is associated with considerable healthcare utilization and economic burden, underscoring the need for effective and safe management strategies.

Over-the-counter nasal decongestants, including xylometazoline and oxymetazoline, are widely used to relieve nasal congestion associated with upper respiratory tract conditions (Alarfaj et al., 2024). These medications provide rapid symptomatic relief by stimulating alpha-adrenergic receptors in the nasal mucosa, producing vasoconstriction, reducing edema, and improving airflow (Alarfaj et al., 2024). Despite their proven effectiveness, clinical guidelines recommend restricting use to a maximum of seven consecutive days to minimize adverse outcomes (Williams and Swift, 2023; Mortuaire et al., 2013). Inappropriate or prolonged use is linked to rebound congestion (rhinitis medicamentosa) and potential systemic side effects such as elevated blood pressure, headache, dizziness, and visual disturbances (Altatan et al., 2025).

In Saudi Arabia, these medications are primarily dispensed through community pharmacies under the supervision of licensed pharmacists, who are expected to provide counseling on appropriate use; however, easy accessibility may allow use without adequate guidance. Several studies have examined the use of nasal decongestants, consistently reporting patterns of misuse, prolonged use, and limited awareness of associated risks (Altatan et al., 2025; Alkalash et al., 2024; Alharthi et al., 2022). However, existing evidence suggests that knowledge does not necessarily translate into appropriate practice, with inappropriate use reported even among individuals with some level of awareness. Most studies have been conducted in specific regions, while national-level evidence remains limited and has primarily focused on prevalence rather than public attitudes and actual practices (Altatan et al., 2025; Alkalash et al., 2024; Alharthi et al., 2022). Moreover, few investigations have examined predictors of appropriate use, highlighting important gaps in understanding the factors influencing safe utilization.

Therefore, this study was designed to address these gaps by focusing specifically on xylometazoline, as it is one of the most widely used topical nasal decongestants globally and is commonly available over the counter (Pintol et al., 2023; Werkhäuser et al., 2022). In Saudi Arabia, its prominence is further supported by data from the Saudi Food and Drug Authority, which indicate a higher number of registered xylometazoline-containing products compared with other nasal decongestants (Saudi Food Drug Authority, 2025). Despite its effectiveness, its widespread availability and ease of access may increase the risk of inappropriate and prolonged use, making it a clinically relevant focus for evaluating public knowledge, attitudes, and practices.

## Methods

### Study design and population

This study employed a cross-sectional survey design to assess the KAP of the general adult population regarding the use of xylometazoline nasal spray. The survey targeted adults aged 18 years and above who were residing in Saudi Arabia at the time of data collection.

### Questionnaire development

The questionnaire was developed following an extensive review of the literature on nasal decongestant sprays, with particular focus on xylometazoline (Alkalash et al., 2024; Al-Rasheedi, 2023; Alyahya et al., 2020). To ensure content validity, clarity, and alignment with the study objectives, the initial draft was reviewed by a panel of experts in clinical pharmacy and public health. The final questionnaire consisted of 47 items divided into four main sections. The first section collected sociodemographic information, including age, gender, region of residence, education level, and smoking status. It also included prior and recent xylometazoline nasal spray use, frequency of use, and the presence of chronic conditions. The second section assessed knowledge about xylometazoline through twelve items covering recommended frequency and duration of use, indications, contraindications, safety in special populations, rebound effects, potential drug interactions, and recognition of common side effects. Each correct response was assigned a score of one, while incorrect or “I do not know” responses were assigned a score of zero, as both indicate a lack of correct knowledge. The total knowledge score ranged from zero to twelve and was classified as poor (0–4), moderate (5–8), or good (9–12). The third section examined participants’ attitudes using five items that explored perceived effectiveness, potential harm, the importance of consulting healthcare professionals, willingness to recommend xylometazoline, and preference for natural alternatives. Positive responses were scored as one and negative or uncertain responses scored as zero, as uncertainty reflects the absence of a clearly positive attitude. Attitude scores were categorized as negative (0–2), moderate (3), or positive (4–5). The final section evaluated practices using six items measured on a four-point scale, where responses were graded as one for best practice, 0.5 for acceptable practice, 0.25 for suboptimal practice, and zero for poor practice. This weighted scoring approach was used to capture varying levels of behavior rather than treating practices as strictly dichotomous (Memon et al., 2015; Gillani et al., 2018). The total practice score ranged from zero to six and was further categorized as poor (0–2), moderate (3–4), or good (5–6).

The questionnaire was originally developed in English and subsequently translated into Arabic, the native language of the target population, to maximize comprehension and accuracy of responses. The English and Arabic versions were cross-checked to ensure consistency. The average time required to complete the survey was approximately 7 minutes.

### Pilot testing

A pilot study was conducted among fifteen participants to assess clarity, feasibility, and completion time. Based on the feedback received, minor revisions were made to improve readability and flow. Cronbach’s alpha test was performed to determine the questionnaire’s reliability. A Cronbach’s alpha of 0.60–0.80 is considered acceptable on a scale of 0–1 (Taber, 2018). The overall Cronbach’s alpha coefficient was 0.766, indicating its reliability.

### Data collection

Data were collected over a 2-month period from May to July 2025 using a self-administrated questionnaire distributed via Google Forms. The sample size was calculated using the Raosoft online calculator (version 2004, Seattle, USA) with a 95% confidence level, 5% margin of error, and 50% response distribution, yielding a target of 385 participants. The survey was pretested online prior to formal distribution to ensure clarity, feasibility, and reliability before formal distribution. A convenience sampling strategy was adopted, and participants were recruited through online dissemination via social media platforms such as WhatsApp. Only respondents who provided informed consent and completed the questionnaire were included in the final analysis.

### Statistical analysis

Descriptive statistics were used to summarize the data: categorical variables were presented as frequencies and percentages, and continuous variables as means and standard deviations. The normality of continuous variables was assessed using the Shapiro-Wilk test. After a normality test was performed on the KAP scores, the data were not normally distributed (Shapiro-Wilk test, p < 0.001). For this reason, the KAP scores were presented using the median and interquartile range (IQR). The chi-square test was applied to assess associations between categorical variables. Spearman correlation analysis was used to assess the relationship between total knowledge, attitude, and practice scores, as these scores were non-normally distributed. The variations between sociodemographic groups in terms of knowledge, attitude, and practice scores were analyzed using the Wilcoxon rank-sum test for variables with two groups or the Kruskal–Wallis rank-sum test for variables with three or more groups.

Binary logistic regression was used to identify independent predictors of high knowledge, positive attitude, and moderate/good practice levels. For knowledge and attitude outcomes, variables were dichotomized by combining poor and moderate categories and comparing them with the highest category. For the practice outcome, moderate and good categories were combined and compared with poor practice due to the small number of participants classified as having good practice. Both crude odds ratios (CORs) and adjusted odds ratios (aORs) with 95% confidence intervals (CIs) were reported. Variables with p < 0.20 in univariate logistic regression were included in the multivariate models. Multicollinearity among independent variables was assessed prior to model fitting, and no significant multicollinearity was detected. The Hosmer–Lemeshow test was used to assess the goodness of fit for the regression models. The results indicated that all models had acceptable fit (knowledge: p = 0.237; attitude: p = 0.747; practice: p = 0.180). A p-value <0.05 was considered statistically significant. Data were analyzed using RStudio (version 4.3.0).

### Ethical considerations

The study protocol was reviewed and approved by the Scientific Research Ethics Committee at Taif University, Saudi Arabia (Approval No. 46-251, dated 16 March 2025). Participation in the study was voluntary. Informed consent was obtained electronically from all participants prior to survey initiation. Anonymity and confidentiality of responses were ensured, and no personally identifiable information was collected.

## Results

### Participants’ demographic characteristics

The study involved a total of 422 participants. A slight majority (53.6%) of the study group consisted of females and almost 43.8% were young adults aged 18–30 years. When it came to education, nearly two-thirds (63.1%) held a bachelor’s degree, followed by 20.6% who had a diploma or below. Regarding geographic distribution, Makkah had the highest percentage of participation (61.6%), followed by Riyadh (10.9%). Most participants were nonsmokers (88.4%). Nearly half of the participants did not report any health conditions (44.3%); in contrast, 26.1% had sinusitis. In terms of xylometazoline use, over two-thirds of participants (69.9%) reported using xylometazoline within the previous 12 months. Approximately 37.4% of respondents reported using xylometazoline less than once the previous year, whereas 41.0% reported using it 1–3 times, 9.7% used it 4–6 times, and 11.8% reported using it more than 6 times over the same 12-month period. One-third of the participants (33.2%) reported not being informed about the potential side effects of xylometazoline by their doctor or pharmacist. Table 1 provides a summary of the participants’ demographic characteristics.

**TABLE 1:** Demographic characteristics of study participants (n = 422).

Characteristics	n (%)
Gender	
Male	196 (46.4)
Female	226 (53.6)
Age (years)	
18–30	185 (43.8)
31–40	111 (26.3)
41–50	67 (15.9)
>50	59 (14.0)
Education level	
Diploma or below	87 (20.6)
Bachelor’s degree	266 (63.1)
Postgraduate studies	69 (16.4)
Residency city/Area	
Makkah	260 (61.6)
Riyadh	46 (10.9)
Asser	36 (8.5)
Eastern	17 (4.0)
Northern borders	16 (3.8)
Madinah	12 (2.8)
Others	35 (8.3)
Smoking status	
Yes	49 (11.6)
No	373 (88.4)
Health conditions reported (*from a predefined list of options)*	
None	187 (44.3)
Sinusitis	110 (26.1)
Chronic allergy	29 (6.9)
Asthma	18 (4.3)
Chronic nasal infections with severe dryness	17 (4.0)
Others	61 (14.4)
Use of xylometazoline spray in past 12 months	
Yes	295 (69.9)
No	127 (30.1)
Annual frequency of xylometazoline spray use	
Less than once a year	158 (37.4)
1–3 times a year	173 (41.0)
4–6 times a year	41 (9.7)
More than 6 times a year	50 (11.8)
Source of information about potential side effects	
I was not informed	140 (33.2)
Explained by the doctor	57 (13.5)
Explained by the pharmacist	88 (20.9)
Explained by both	43 (10.2)
I do not remember	94 (22.3)

### Levels of knowledge, attitudes, and practices toward xylometazoline drug use

Participants showed different levels of knowledge, Attitudes, and Practices concerning xylometazoline use. The median knowledge score, which could range from 0 to 12, was 9.00, indicating a high level of knowledge. Just over half (51.4%) demonstrated good knowledge, with a large portion (46.5%) showing moderate knowledge, and only a small minority (2.1%) having poor knowledge. Regarding attitudes, the median attitudes score of 4.00 (possible range: 1–5) indicated favorable attitude. About 55.0% of participants held positive attitude, while 31.0% had a moderate attitude, and 14% had a negative attitude. However, when it came to actual practices, the median practice score was 2.25 (with a possible range of 0–6). The results showed an opposite trend, with a significant proportion of participants (46.9%) demonstrating moderate practices, 44.3% reporting poor practices, and only 8.8% exhibiting good practices. Table 2 presents the detailed distribution of participants’ knowledge, attitude, and practice levels regarding xylometazoline use.

**TABLE 2:** Distribution of knowledge, attitude, and practices levels toward xylometazoline drug use among participants.

Domain	Level	(n)	(%)	Median (IQR)
Knowledge	Poor (0–4)	9	2.1	9.00 (3.00)
	Moderate (5–8)	196	46.5	
	Good (9–12)	217	51.4	
Attitude	Negative (0–2)	59	14.0	4.00 (1.00)
	Moderate (3)	131	31.0	
	Positive (4–5)	232	55.0	
Practice	Poor (0–2)	187	44.3	2.25 (1.75)
	Moderate ( >2 –4)	198	46.9	
	Good ( >4 –6)	37	8.8	

### Association between knowledge level, attitudes, and practice toward xylometazoline use

Participants with good knowledge of xylometazoline were significantly more likely to have a positive attitude toward its use (62.2%), with 30.4% showing an average attitude and only 7.4% had a negative attitude. Conversely, for those with low knowledge, attitudes varied. Over half (55.6%) still demonstrated a positive attitude, yet about one-third (33.3%) had a negative attitude, with only 11.1% showing a moderate attitude. A statistically significant relationship was observed between categorized knowledge levels and attitude levels (χ^2^, p < 0.001), suggesting these two factors are not independent. Also, a significant relationship was found between participants’ categorized knowledge levels and their practice levels (χ^2^, p = 0.018).

Participants with a positive attitude toward xylometazoline use were significantly more likely to exhibit moderate practices (50.4%). Within this group, 38.4% showed poor practices, and only 11.2% demonstrated good practices. Similarly, among those with a negative attitude, the majority (59.3%) still displayed poor practices. A considerable portion (30.5%) had moderate practices, while only 10.2% showed good practices. A significant correlation (χ^2^, p = 0.005) was found between categorized attitude levels and practice levels. Table 3 show the distribution of attitude and practice levels by knowledge and attitude categories.

**TABLE 3:** Distribution of attitude and practice levels by knowledge and attitude categories among participants.

(a) Attitude distribution by knowledge category				
Knowledge category	Negative attitude	Average attitude	Positive attitude	P Value
Poor knowledge	3 (33.3%)	1 (11.1%)	5 (55.6%)	**<0.001**
Moderate knowledge	40 (20.4%)	64 (32.7%)	92 (46.9%)	
Good knowledge	16 (7.4%)	66 (30.4%)	135 (62.2%)	

(b) Practice distribution by knowledge category				
​	Poor practice	Moderate practice	Good practice	P Value
Poor knowledge	8 (88.9%)	1 (11.1%)	0 (0.0%)	**0.018**
Moderate knowledge	95 (48.5%)	83 (42.3%)	18 (9.2%)	
Good knowledge	84 (38.7%)	114 (52.5%)	19 (8.8%)	

(c) Practice distribution by attitude category				
​	Poor practice	Moderate practice	Good practice	P Value
Negative attitude	35 (59.3%)	18 (30.5%)	6 (10.2%)	**0.005**
Average attitude	63 (48.1%)	63 (48.1%)	5 (3.8%)	
Positive attitude	89 (38.4%)	117 (50.4%)	26 (11.2%)	

### Correlations between knowledge, attitude, and practice scores toward xylometazoline use

The correlation analysis revealed weak but statistically significant positive associations between knowledge, attitude, and practice toward xylometazoline use (Table 6). Knowledge was positively correlated with both practice (rho = 0.199, p < 0.001) and attitude (rho = 0.206, p < 0.001). Similarly, a weak positive correlation was observed between attitude and practice (rho = 0.199, p < 0.001).

**FIGURE 1:**
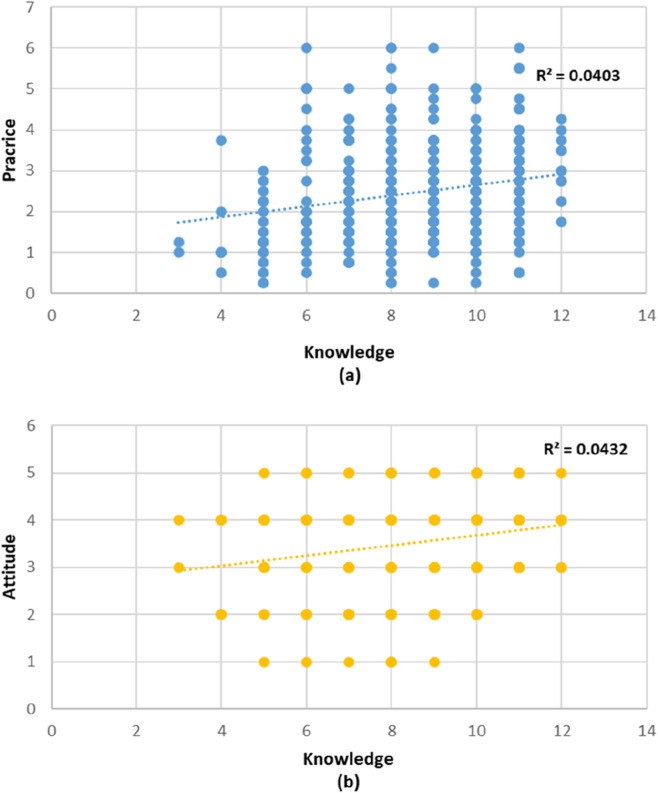
Scatterplots showing the relationships between knowledge and practice **(a)** and knowledge and attitude **(b)** toward Xylometazoline use.

The scatterplots illustrate these relationships. Figure 1A shows the association between knowledge and practice, with a slight upward trend in practice scores as knowledge increases (*R* ^2^ = 0.0403). Figure 1B demonstrates the relationship between knowledge and attitude, also showing a weak positive association (*R* ^2^ = 0.0432). Indicating minimal associations between the variables.

### Predictors of high knowledge toward xylometazoline use

Logistic regression analysis identified several independent predictors of good knowledge toward xylometazoline use (Table 4). Compared to participants aged ≤30 years, those aged 31–40 years (aOR = 0.57, 95% CI: 0.34–0.95, p = 0.031), 41–50 years (aOR = 0.39, 95% CI: 0.21–0.73, p = 0.003), and >50 years (aOR = 0.35, 95% CI: 0.18–0.67, p = 0.001) had significantly lower odds of demonstrating good knowledge. Higher educational attainment was positively associated with good knowledge; participants with a bachelor’s degree (aOR = 1.51, 95% CI: 1.03–2.22, p = 0.033) or postgraduate studies (aOR = 2.78, 95% CI: 1.42–5.45, p = 0.003) were more likely to report good knowledge compared to those with diploma or less. Participants from “other” regions (aOR = 0.42, 95% CI: 0.20–0.91, p = 0.028) had significantly lower odds of good knowledge, while those with chronic nasal infections and severe dryness had markedly higher odds (aOR = 6.49, 95% CI: 1.70–24.79, p = 0.006).

**TABLE 4:** Logistic regression analysis of independent factors for knowledge of participants.

Risk factors	Good level of knowledge (%)	COR (95% CI)	*P* Value	aOR (95% CI)	P Value
*Age*					
≤30	111 (60.0%)	1	​	1	​
31–40	56 (50.5%)	1.02 (0.70–1.48)	0.924	0.57 (0.34–0.95)	0.031
41–50	27 (40.3%)	0.68 (0.41–1.10)	0.115	0.39 (0.21–0.73)	0.003
More than 50	23 (39.0%)	0.64 (0.38–1.08)	0.093	0.35 (0.18–0.67)	0.001
*Gender*					
Male	100 (51.0%)	1	​	​	​
Female	117 (51.8%)	1.07 (0.83–1.39)	0.595	​	​
*Education level*					
Diploma degree or less	34 (39.1%)	1	​	1	​
Bachelor’s degree	143 (53.8%)	1.16 (0.91–1.48)	0.221	1.51 (1.03–2.22)	0.033
Postgraduate studies	40 (58.0%)	1.38 (0.86–2.23)	0.187	2.78 (1.42–5.45)	0.003
*Residence*					
Makkah	134 (51.5%)	1	​	1	​
Riyadh	28 (60.9%)	1.56 (0.86–2.82)	0.144	1.40 (0.71–2.75)	0.327
Asser	15 (41.7%)	0.71 (0.37–1.39)	0.320	0.79 (0.37–1.68)	0.535
Eastern	12 (70.6%)	2.40 (0.85–6.82)	0.100	2.57 (0.84–7.88)	0.100
Northern borders	9 (56.3%)	1.29 (0.48–3.45)	0.618	1.28 (0.43–3.83)	0.654
Madinah	6 (50.0%)	1.00 (0.32–3.10)	1.000	0.64 (0.18–2.23)	0.484
Others	13 (37.1%)	0.59 (0.30–1.17)	0.133	0.42 (0.20–0.91)	0.028
*Smoking status*					
Yes	26 (53.1%)	1	​	​	​
No	191 (51.2%)	1.13 (0.65–1.98)	0.668	​	​
*Health conditions reported*					
None	92 (49.2%)	1	​	1	​
Sinusitis	61 (55.5%)	1.25 (0.86–1.81)	0.254	1.59 (0.96–2.63)	0.072
Chronic allergy	17 (58.6%)	1.42 (0.68–2.97)	0.356	1.95 (0.83–4.55)	0.124
Asthma	8 (44.4%)	0.80 (0.32–2.03)	0.638	0.73 (0.27–1.97)	0.528
Chronic nasal infections with severe dryness	14 (82.4%)	4.67 (1.34–16.24)	0.015	6.49 (1.70–24.79)	0.006
Others	25 (41.0%)	0.69 (0.42–1.16)	0.161	1.01 (0.53–1.92)	0.982
*Annual frequency of Xylometazoline spray use*					
Less than once a year	75 (47.5%)	1	​	​	​
1–3 times a year	92 (54.2%)	1.14 (0.84–1.53)	0.403	​	​
4–6 times a year	24 (58.5%)	1.41 (0.76–2.63)	0.277	​	​
More than 6 times a year	26 (52.0%)	1.08 (0.62–1.89)	0.777	​	​
*Source of information about potential side effects*					
I was not informed	75 (53.6%)	1	​	1	​
Explained by the doctor	27 (47.4%)	0.90 (0.54–1.51)	0.691	0.77 (0.41–1.43)	0.404
Explained by the pharmacist	53 (60.2%)	1.51 (0.99–2.32)	0.057	1.31 (0.76–2.27)	0.329
Explained by both	20 (46.5%)	0.87 (0.48–1.58)	0.648	0.66 (0.32–1.37)	0.269
I do not remember	42 (44.7%)	0.81 (0.54–1.21)	0.303	0.66 (0.39–1.12)	0.126

### Predictors of moderate/Good practice toward xylometazoline use

Independent predictors of moderate/good practice are presented in Table 5. Female participants were significantly more likely to report moderate/good practice than males (aOR = 1.69, 95% CI: 1.13–2.53, p = 0.011). Educational attainment was a strong predictor, with bachelor’s degree (aOR = 2.22, 95% CI: 1.48–3.32, p < 0.001) and postgraduate studies (aOR = 1.84, 95% CI: 1.02–3.32, p = 0.044) associated with higher odds of moderate/good practice. Regarding health conditions, sinusitis (aOR = 1.85, 95% CI: 1.07–3.20, p = 0.029) and chronic nasal infections with severe dryness (aOR = 6.29, 95% CI: 1.72–22.97, p = 0.005) were significant predictors of moderate/good practice. Frequency of spray use was inversely associated with practice quality; compared to those using the spray less than once a year, participants using it more frequently had significantly lower odds of moderate/good practice (1–3 times: aOR = 0.37, p < 0.001; 4–6 times: aOR = 0.18, p < 0.001; >6 times: aOR = 0.05, p < 0.001).

**TABLE 5:** Logistic regression analysis of independent factors against Practice of participants.

Risk factors	Moderate/Good level of practice (%)	COR (95% CI)	*P* Value	aOR (95% CI)	P Value
*Age*					
18–30	107 (57.8%)	1	​	​	​
31–40	63 (56.8%)	1.31 (0.90–1.91)	0.156	​	​
41–50	36 (53.7%)	1.16 (0.72–1.88)	0.542	​	​
More than 50	29 (49.2%)	0.97 (0.58–1.61)	0.896	​	​
*Gender*					
Male	99 (50.5%)	1	​	1	​
Female	136 (60.2%)	1.51 (1.16–1.97)	0.002	1.69 (1.13–2.53)	0.011
*Education level*					
Diploma degree or less	41 (47.1%)	1	​	1	​
Bachelor’s degree	158 (59.4%)	1.46 (1.15–1.87)	0.002	2.22 (1.48–3.32)	<0.001
Postgraduate studies	36 (52.2%)	1.09 (0.68–1.75)	0.718	1.84 (1.02–3.32)	0.044
*Residence*					
Makkah	140 (53.8%)	1	​	​	​
Riyadh	28 (60.9%)	1.56 (0.86–2.81)	0.144	​	​
Asser	23 (63.9%)	1.77 (0.90–3.49)	0.100	​	​
Eastern	10 (58.8%)	1.43 (0.54–3.75)	0.469	​	​
Northern borders	9 (56.3%)	1.29 (0.48–3.45)	0.618	​	​
Madinah	9 (75%)	3.00 (0.81–11.08)	0.099	​	​
Others	16 (45.7%)	0.84 (0.43–1.64)	0.613	​	​
*Smoking status*					
Yes	20 (40.8%)	1	​	​	​
No	215 (57.6%)	0.69 (0.39–1.22)	0.201	​	​
*Health conditions reported*					
None	106 (56.7%)	1	​	1	​
Sinusitis	61 (55.5%)	1.25 (0.86–1.81)	0.254	1.85 (1.07–3.20)	0.029
Chronic allergy	18 (62.1%)	1.64 (0.77–3.47)	0.198	2.53 (0.99–6.49)	0.054
Asthma	10 (55.6%)	1.25 (0.49–3.17)	0.638	1.21 (0.43–3.37)	0.720
Chronic nasal infections with severe dryness	11 (64.7%)	1.83 (0.68–4.957)	0.232	6.29 (1.72–22.97)	0.005
Others	29 (47.5%)	0.91 (0.55–1.50)	0.701	0.72 (0.40–1.31)	0.282
*Annual frequency of Xylometazoline spray use*					
Less than once a year	117 (74.1%)	1	​	1	​
1–3 times a year	91 (52.6%)	1.11 (0.82–1.50)	0.494	0.37 (0.24–0.59)	<0.001
4–6 times a year	17 (41.5%)	0.71 (0.38–1.32)	0.277	0.18 (0.08–0.39)	<0.001
More than 6 times a year	10 (20.0%)	0.25 (0.13–0.50)	<0.001	0.05 (0.02–0.11)	<0.001
Source of information about potential side effects					
I Was not informed	81 (57.9%)	1	​	​	​
Explained by the doctor	26 (45.6%)	0.84 (0.50–1.41)	0.508	​	​
Explained by the pharmacist	52 (59.1%)	1.44 (0.94–2.21)	0.090	​	​
Explained by both	27 (62.8%)	1.69 (0.91–3.13)	0.097	​	​
I do not remember	49 (52.1%)	1.09 (0.73–1.63)	0.680	​	​

### Predictors of positive attitude toward xylometazoline use

As shown in Table 6, participants aged >50 years had significantly lower odds of a positive attitude toward xylometazoline use (aOR = 0.51, 95% CI: 0.27–0.96, p = 0.037). Regional variation was observed, with those from “other” regions reporting higher odds of positive attitude compared with Makkah (aOR = 2.50, 95% CI: 1.20–5.21, p = 0.014). Among health conditions, sinusitis (COR = 1.62, 95% CI: 1.10–2.38, p = 0.014) and chronic nasal infections with severe dryness (COR = 3.25, 95% CI: 1.06–9.97, p = 0.039) were associated with positive attitude, although these associations were not significant after adjustment.

**TABLE 6:** Logistic regression analysis of independent factors for attitude of participants.

Risk factors	Positive level of attitude (%)	COR (95% CI)	*P* Value	aOR (95% CI)	P Value
*Age*					
18–30	110 (59.5%)	1	​	1	​
31–40	58 (52.3%)	1.09 (0.75–1.59)	0.635	0.87 (0.55–1.37)	0.548
41–50	41 (61.2%)	1.58 (0.97–2.58)	0.069	1.24 (0.69–2.25)	0.476
More than 50	23 (39.0%)	0.64 (0.38–1.08)	0.093	0.51 (0.27–0.96)	0.037
*Gender*					
Male	103 (52.6%)	1	​	1	​
Female	129 (57.1%)	1.33 (1.02–1.73)	0.034	1.32 (0.91–1.90)	0.142
*Education level*					
Diploma degree or less	41 (47.1%)	1	​	​	​
Bachelor’s degree	153 (57.5%)	1.35 (1.06–1.73)	0.015	​	​
Postgraduate studies	38 (55.1%)	1.23 (0.76–1.97)	0.400	​	​
*Residence*					
Makkah	143 (55.0%)	1	​	​	​
Riyadh	21 (45.7%)	0.84 (0.47–1.50)	0.556	​	​
Asser	19 (52.8%)	1.12 (0.58–2.15)	0.739	​	​
Eastern	9 (52.9%)	1.13 (0.43–2.92)	0.808	​	​
Northern borders	10 (62.5%)	1.67 (0.61–4.59)	0.323	​	​
Madinah	5 (41.7%)	0.71 (0.23–2.25)	0.566	​	​
Others	21 (71.4%)	2.50 (1.20–5.21)	0.014	​	​
*Smoking status*					
Yes	27 (55.1%)	1	​	​	​
No	205 (55.0%)	1.23 (0.70–2.16)	0.48	​	​
*Health Conditions Reported*					
None	106 (56.7%)	1	​	​	​
Sinusitis	68 (61.8%)	1.62 (1.10–2.38)	0.014	​	​
Chronic allergy	13 (44.8%)	0.81 (0.39–1.69)	0.578	​	​
Asthma	8 (44.4%)	0.80 (0.32–2.03)	0.638	​	​
Chronic nasal infections with severe dryness	13 (76.5%)	3.25 (1.06–9.97)	0.039	​	​
Others	24 (39.3%)	0.65 (0.39–1.08)	0.099	​	​
*Annual frequency of Xylometazoline spray use*					
Less than once a year	88 (55.7%)	1	​	1	​
1–3 times a year	95 (54.9%)	1.22 (0.90–1.64)	0.197	0.96 (0.63–1.45)	0.837
4–6 times a year	16 (39.0%)	0.64 (0.34–1.20)	0.163	0.51 (0.25–1.04)	0.063
More than 6 times a year	33 (66.0%)	1.94 (1.08–3.49)	0.026	1.36 (0.66–2.79)	0.402
*Source of information about potential side effects*					
I Was not informed	74 (52.9%)	1	​	1	​
Explained by the doctor	37 (64.9%)	1.85 (1.07–3.19)	0.027	1.60 (0.85–3.02)	0.145
Explained by the pharmacist	53 (60.2%)	1.51 (0.99–2.32)	0.057	1.59 (0.95–2.67)	0.076
Explained by both	26 (60.5%)	1.53 (0.83–2.82)	0.173	1.33 (0.66–2.69)	0.425
I do not remember	42 (44.7%)	0.81 (0.54–1.21)	0.303	0.79 (0.47–1.31)	0.353

## Discussion

This study examined public KAP toward xylometazoline use and identified sociodemographic and clinical predictors of appropriate utilization. Xylometazoline use within the past 12 months was highly prevalent among participants. While knowledge and attitudes toward the medication were generally favorable, actual practices were weaker, with only a small proportion adhering fully to recommended usage guidelines. Significant associations were observed among knowledge, attitudes, and practices, indicating that higher knowledge was linked with more positive attitudes and, to some extent, safer practices. Younger age and higher education emerged as strong predictors of better knowledge, whereas women and those with chronic nasal conditions demonstrated safer practices. By contrast, older participants were less likely to report positive attitudes. These findings highlight a clear gap between awareness and behavior, emphasizing the importance of targeted health education and pharmacist-led counseling to promote safe and effective use of xylometazoline.

The prevalence of xylometazoline use in this study was high, with 70% of participants reporting use in the past year, consistent with the 68.5% prevalence of nasal decongestant use reported among Saudi individuals (Alharthi et al., 2022). This high utilization may be attributed to the low cost, over-the-counter accessibility, and frequent prescribing of these medications (Rameshkumar et al., 2022). National and international studies have similarly reported substantial misuse, often linked to limited awareness of potential risks (Salama et al., 2025).

In the present study, approximately half of participants reported limiting use to 3 days or less, aligning with recommended durations. Clinical guidance advises restricting use to no more than three to five consecutive days, as prolonged use is associated with rhinitis medicamentosa (Mortuaire et al., 2013; Lenz et al., 2011). This condition is thought to result from receptor downregulation following sustained alpha-adrenergic stimulation, leading to diminished vasoconstriction and subsequent rebound vasodilation (Mösges et al., 2017). Although different concentrations are available for different populations (e.g., 0.05% for pediatric use and 0.1% for adults), the risk of rhinitis medicamentosa is similar across concentrations, with duration of use remaining the primary determinant (Chi, 2023). Additionally, although administered topically, some systemic absorption may occur; therefore, caution is warranted in vulnerable populations, particularly individuals with cardiovascular conditions, due to potential sympathomimetic effects. Comparable findings were reported in a 2022 national study, where 70.8% of users limited their use to less than 5 days (Alharthi et al., 2022). However, studies in developed countries indicate that 31.9%–49% of users exceed the recommended duration (Mehuys et al., 2014; Russo et al., 2023; Kariri et al., 2025).

Regarding knowledge, attitudes, and practices, over half of participants demonstrated good knowledge, and more than half expressed positive attitudes. However, only 8.8% exhibited good practices, while 44.3% showed poor practices. These results demonstrate that awareness and attitudes do not always translate into safe behavior. The weak but statistically significant correlations among knowledge, attitudes, and practices further support this observation; however, their strength was minimal, indicating limited associations and explanatory power. This suggests that additional factors may influence behavior. This is consistent with previous findings linking health literacy with treatment adherence, though often with modest effects (Kariri et al., 2025; Miller, 2016). In contrast, some Saudi studies have reported poorer KAP outcomes (Al-Rasheedi, 2023), which may reflect differences in study settings, populations, or methods of assessment.

The analysis of predictors provides further insights. Younger adults were significantly more likely to report good knowledge, but also had a higher prevalence of use compared with older age groups. Higher educational attainment was associated with better knowledge and safer practices, confirming prior evidence that education enhances medication awareness and health-seeking behaviors (Chui et al., 2017; Bekele et al., 2020). Women were more likely than men to demonstrate good practice, and participants with sinusitis or chronic nasal conditions also reported safer practices, likely reflecting greater exposure to healthcare and medical advice. Conversely, older adults were less likely to report positive attitudes, which may reflect generational differences in perceptions of medication use. Geographic variation was evident, with participants from regions outside Makkah less likely to have good knowledge compared to those residing in Makkah. This difference may be partly attributable to the large proportion of participants recruited from Makkah, which may have influenced the overall statistical comparison. However, this geographic imbalance may limit the representativeness of the sample and should be considered when interpreting the findings. Additionally, some estimates, particularly those observed in smaller subgroups, should be interpreted with caution, as they may be unstable and potentially overestimated.

### Implications for practice and public health

This study highlights the need for targeted educational interventions addressing the gap between knowledge and practice. While knowledge is relatively high, a lack of professional guidance was observed, with one-third of participants reporting no counseling from healthcare providers, despite the widespread availability of community pharmacists. This finding suggests missed opportunities for pharmacist-led interventions to educate patients on appropriate duration of use, potential risks, and safe medication practices. Pharmacists should play a stronger role in patient education, given their accessibility in community settings, particularly in addressing misconceptions that over-the-counter medications are inherently safe. Strengthening counseling practices could mitigate the risks of inappropriate use, including dependency and rebound congestion.

From a public health perspective, the high prevalence of xylometazoline use indicates that nasal decongestants are commonly relied upon in the population. This highlights the need for stricter regulatory oversight and public campaigns to reduce misuse and prevent potential complications such as rhinitis medicamentosa. The positive correlations among knowledge, attitude, and practice further suggest that integrated strategies promoting awareness and behavior modification could yield substantial benefits. However, from a regulatory perspective, xylometazoline is currently available as an over-the-counter medication in Saudi Arabia, which may contribute to its widespread use and potential misuse. The findings of this study support consideration of additional regulatory measures, such as enhanced warning labels, restrictions on package size, or increased pharmacist oversight, to promote appropriate use and reduce the risk of adverse outcomes.

### Strengths and limitations

This is the first study to assess the knowledge, attitudes, and practices toward xylometazoline use across Saudi Arabia, providing insights from a broad sample of the population rather than a fully representative national perspective. The relatively large sample size enhances the generalizability of the findings, while the use of a structured and validated questionnaire, developed through literature review and expert evaluation, strengthens methodological rigor. The tool demonstrated acceptable internal consistency, and the translation with back-translation ensured accuracy in both English and Arabic versions, reducing language-related bias.

Despite these strengths, some limitations should be acknowledged. The cross-sectional design prevents causal inferences, and convenience sampling through online platforms may have favored younger, more educated, and digitally literate participants, potentially overestimating knowledge levels and limiting the representativeness of the sample, with a risk of selection bias. Reliance on self-reported data may also introduce recall and social desirability bias, particularly regarding the frequency, duration, and appropriateness of xylometazoline use. Additionally, although overall reliability was assessed and found to be acceptable, reliability was not evaluated separately for the knowledge, attitude, and practice domains, which may limit the interpretation of internal consistency across individual sections. Furthermore, although participants reported whether they received information from healthcare providers, the study did not assess the quality or effectiveness of counseling, limiting interpretation of its impact. Finally, although the tool was validated, the self-administered nature of the tool may still allow for measurement error.

### Future research

Future studies should incorporate qualitative approaches to explore the behavioral and cultural determinants that contribute to poor practices despite adequate knowledge. Such insights would help uncover patient beliefs, social norms, and healthcare communication gaps that quantitative data alone cannot fully capture. In addition, interventional studies evaluating the impact of pharmacist-led counseling, digital health education programs, and regulatory restrictions on the purchase of nasal decongestants are needed to generate actionable evidence for clinical and policy decision-making. Comparative investigations across different regions of Saudi Arabia and international contexts would further contextualize these findings, allowing for the development of tailored interventions that address both local and global patterns of nasal decongestant use. Future studies should also evaluate how the source of medication influences the provision and quality of counseling, to better understand its impact on patient behavior and safe medication use.

## Conclusion

This study revealed that knowledge and attitudes toward xylometazoline were generally favorable, yet appropriate practice was uncommon and only weakly related to knowledge or attitudes. Predictors such as age, education, gender, and health conditions highlight priority groups for targeted interventions. Addressing the identified knowledge–practice gap through structured counseling, regulatory measures, and public health education campaigns is essential to mitigate the risks of inappropriate use patterns and promote safer use of nasal decongestants.

## Data Availability

The raw data supporting the conclusions of this article will be made available by the authors, without undue reservation.
